# A Novel High-Throughput Sample-in-Result-Out Device for the Rapid Detection of Viral Nucleic Acids

**DOI:** 10.3390/bios14110549

**Published:** 2024-11-13

**Authors:** Fangning Wang, Fei Hu, Yunyun Zhang, Xichen Li, Qin Ma, Xincheng Wang, Niancai Peng

**Affiliations:** State Key Laboratory for Manufacturing Systems Engineering, School of Instrument Science and Techonology, Xi’an Jiaotong University, Xi’an 710054, China; fangningwang@stu.xjtu.edu.cn (F.W.); zhangyunyun0405@163.com (Y.Z.); lxc123456@stu.xjtu.edu.cn (X.L.); maqinaaa@163.com (Q.M.); engineerwangxc@163.com (X.W.); pnc@mail.xjtu.edu.cn (N.P.)

**Keywords:** CRISPR-Cas12a, CRISPR-Cas13a, RPA, nucleic acid detection, two-gene detection

## Abstract

Clustered regularly interspaced short palindromic repeats (CRISPR) molecular diagnostic technology is one of the most reliable diagnostic tools for infectious diseases due to its short reaction time, high sensitivity, and excellent specificity. However, compared with fluorescent polymerase chain reaction (PCR) technology, CRISPR molecular diagnostic technology lacks high-throughput automated instrumentation and standardized detection reagents for high sensitivity, limiting its large-scale clinical application. In this study, a high-throughput automated device was developed by combining reagent lyophilization, extraction-free technology, and a one-pot consumable system. This innovative approach enabled the rapid sample-in-result-out detection of 48 samples in 25 min and demonstrated high sensitivity and specificity for the qualitative analysis of clinical samples. The obtained results show that the detection limit of the designed system for African swine fever virus (ASFV) is 0.5 copies/μL. As a proof concept, a single-tube dual-target nucleic acid detection method was developed, achieving a detection limit of 5 copies/μL for the *ORF1ab* and *N* genes of severe acute respiratory syndrome coronavirus-2 (SARS-CoV-2) within 45 min. The method is highly specific, reliable, and stable, providing a feasible solution for the clinical application of CRISPR nucleic acid detection technology.

## 1. Introduction

Infectious diseases caused by pathogens significantly impact human life, health, and socioeconomic development [1]. The global spread of pathogens, such as the Ebola virus [2], Zika virus [3], and SARS-CoV-2 has significantly threatened public health worldwide [4]. Therefore, to enhance early infection screening accuracy and the subsequent isolation and treatment effectiveness, the focus of modern-day research has shifted to developing high-sensitivity, -specificity, and -throughput molecular diagnostic technology. This transformation raised the bar for detection technologies, creating an urgent demand for high-sensitivity, rapid-detection, low-cost, high-throughput, automated “sample-in, result-out” nucleic acid testing products [5].

Currently, the quantitative real-time fluorescence PCR method is considered the “gold standard” for the diagnosis of many pathogens due to its high sensitivity and specificity [6]. However, this method is often costly and requires complex and sophisticated instruments and trained personnel [7]. Several isothermal amplification methods have also emerged recently, such as loop-mediated isothermal amplification (LAMP) [8], recombinase polymerase amplification (RPA) [9], and rolling circle amplification (RCA) [10]. Although these methods do not need variable temperature control and provide high amplification efficiency, their detection specificity and sensitivity often do not reach the PCR level.

The CRISPR-Cas system has gradually gained public attention and has been widely used in genetic engineering, gene regulation, gene screening, and nucleic acid detection in recent years [11]. The CRISPR system employs guide RNA (gRNA) sequences encoded in the CRISPR locus to direct Cas endonuclease activity, recognizing and cleaving specific foreign nucleic acid sequences [12]. CRISPR-based nucleic acid assays have high specificity. Once the target is bound to the CRISPR-crRNA complex, the cleavage activity of the Cas enzyme is activated, which nonspecifically cleaves the ssDNA or ssRNA reporter in the system, resulting in a detectable fluorescence signal. Researchers have combined nucleic acid amplification with CRISPR-Cas to detect viral nucleic acids, such as SHERLOCK (specific high-sensitivity enzymatic reporter unlocking) [13], DETECTR (DNA endonuclease-targeted CRISPR trans reporter) [14], and HOLMES (one-hour low-cost multipurpose highly efficient system) [15]. These methods have an overall detection limit of up to aM by introducing the isothermal amplification method, but the detection time is in the range of 1–2 h. Since then, a variety of nucleic acid detection methods based on isothermal amplification and CRISPR-Cas have been reported. Wang et al. [16] developed CRISPR/Cas12a-NER (CRISPR/Cas12a-based assay with a naked-eye readout) to detect SARS-CoV-2. The designed assay uses RT-RAA to amplify the target, shortening the detection time from 120 min to 45 min and enhancing the detection sensitivity to 10 copies/μL. Lin et al. [17] established an all-in-one RPA-Cas12a method (CORDSv2), which achieved ultra-sensitive detection of multiple targets, shortened the detection time to 40 min, and improved the detection limit to 0.6 copies/μL. All of these methods use nucleic acids after extraction by the magnetic bead method, and their extraction steps are time-consuming and complex.

In the recent development of CRISPR technology, extraction-free technology [18,19,20,21,22], reagents lyophilization [21,23,24,25,26], and a variety of one-pot assays to avoid contamination [27,28,29,30,31] have been proposed to minimize experimental complexity. HUDSON (heating unextracted diagnostic samples to obliterate nucleases) employs chemical reduction and/or heat denaturation to lyse pathogens and inactivate sample nucleases within 30 min [22]. This protocol time can be shortened to <10 min by modifying the lysis buffer conditions. A more typical extraction-free technique is used for sPAMC (for suboptimal PAM (protospacer adjacent motifs) of Cas12a-based test), which was proposed by Lu et al. [18], which allows for the detection of the SARS-CoV-2 RNA in 15 min using unextracted samples. Unextracted samples are lysed at 95 °C for 5–10 min with an equal volume of lysis buffer. Further, Arizti-Sanz et al. [21] reported SHINEv.2 (streamlined highlighting of infections to navigate epidemics, version 2), incorporating ambient-temperature sample processing and lyophilized testing reagents. This method greatly simplifies reagent transport and storage and reduces the overall complexity of the assay. In this method, clinical samples are mixed with 5× FastAmp lysis reagent (Intact Genomics, St. Louis, MI, USA) with 5% RNase inhibitor and incubated at ambient temperature for 5 min. Reagent lyophilization is performed by flash-freezing the master mix, except for magnesium acetate, in liquid nitrogen and lyophilizing overnight. For one-pot assays, studies have examined methods that perform sequential or simultaneous nucleic acid amplification and CRISPR reactions in a single tube due to their ability to streamline assay performance and reduce the risk of contamination [32], for example, the use of gRNAs targeting suboptimal PAM sequences, photoactivation, and optimizing the composition of the buffer, but these methods increase the complexity of the method and have reduced sensitivity. Our team [28] previously designed a tube-in-tube consumable structure, where the outer and inner tubes are combined into one. The inner tube contains two hydrophobic holes for nucleic acid amplification reaction. The CRISPR detection reagent is prestored in the inner tube. After completion of the amplification reaction, the products are transferred to the outer tube via hydrophobic holes by centrifugation. This design shortens the detection time and improves detection sensitivity.

In conclusion, the current research on molecular diagnostic techniques based on the CRISPR-Cas system is relatively mature, yet there are still several challenges in their practical application. First, most of the current research remains in the laboratory stage, lacking standardized instruments and experimental methods, which restrict the clinical application of the technology. Most of the methods developed in the laboratory have a throughput of less than 10 in a single assay. Second, the sensitivity of the reported assays varies widely, ranging from 10^4^ copies/μL to 0.5 copies/μL [33,34,35]. Moreover, different reagent systems cause large interlaboratory reproducibility errors. Some highly sensitive methods have complicated reagent systems, adding obstacles to their practical application.

**Figure 1:**
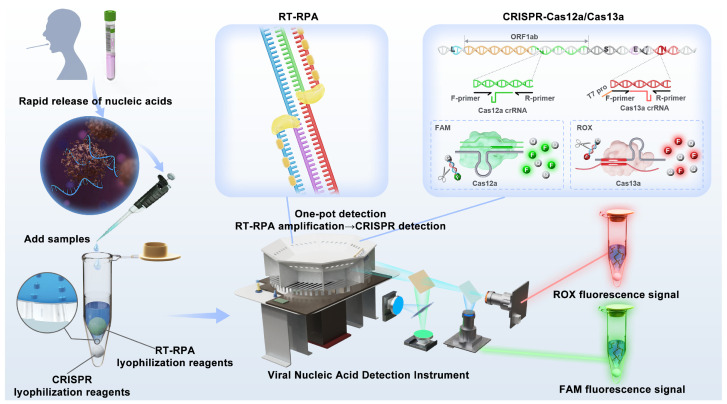
Schematic of CRISPR-based high-throughput sample-in-result-out viral nucleic acid assays. The device integrates heating, centrifugation, optical detection, data analysis, and result output functions. The detection process includes treating nasopharyngeal swabs with sample release reagents, adding the mixture to the inner tube of the reaction tube containing RT-RPA-lyophilized spheres, and centrifuging the amplification products to mix with CRISPR-lyophilized reagents after completing the RT-RPA reaction to participate in the CRISPR-Cas reaction. The optical detection channel has two channels, FAM and ROX, which enable simultaneous dual-target detection in a single tube.

In this study, we aimed to address the above challenges by combining extraction-free technology, reagent lyophilization, RPA, and CRISPR detection. The required reagents for amplification and CRISPR reactions are premixed and lyophilized, and they can be easily reconstituted by adding buffer or target analytes. These lyophilized reagents enable easy, on-site deployment and a longer reagent shelf-life. A high-throughput sample-in-result-out viral nucleic acid detection device was designed and developed, capable of simultaneously detecting 48 samples in 20 min. This device employs a new pathogen nucleic acid detection method with a detection limit of up to 0.5 copies/μL for ASFV. The designed method was also expanded and applied to the CRISPR-Cas12a/Cas13a orthogonal detection system. Figure 1 illustrates the working mechanism and principle of the proposed method. To the best of our knowledge, this method is convenient and reported for the first to realize high-throughput “sample-in, result-out” nucleic acid detection. Also, the method can effectively lower the threshold for the rapid on-site screening of nucleic acid populations and provide a technological reserve for the prevention and control of infectious diseases in the future.

## 2. Materials and Methods

### 2.1. Materials

All primers were synthesized by Sangon Biotech Co., Ltd. (Shanghai, China). The specific sequences of the primers and crRNAs are detailed in Table 1. An RNA Constant Temperature Rapid Amplification Kit (Basic) was purchased from Amp-future Biotech Co., Ltd. (Changzhou, China). Ribonuclease inhibitor and Ribonuclease H (RNase H) were obtained from TaKaRa Biologicals (Dalian, China). Ribonucleotide mixtures (rNTP) and 10×NEB r2.1 buffer were provided by New England Biolabs (Beijing, China). CRISPR RNA (Cas12a-crRNA and Cas13a-crRNA), LbaCas12a, Cas12a buffer, LbuCas13a, LbuCas13a buffer, T7 RNA polymerase, fluorescence quencher-labeled single-stranded DNA, and single-stranded RNA probes were supplied by Bio-Lifesci Co., Ltd. (Guangzhou, China). qPCR diagnostic kits and nucleic acid extraction kits (including magnetic bead, lysis, washing, and elution solutions) were purchased from Xi’an Tianlong Technology Co., Ltd. (Xi’an, China). Reagent lyophilized beads were customized by Tolo Biotech (Shanghai, China). Distilled deionized water (ddH_2_O) was used in all the experiments. All reagents were of analytical grade and were used directly without further purification. DNA reference materials for the African swine fever virus (ASFV), Pseudorabies virus (RPV), and Marek’s disease virus (MDV) were purchased from the Research and Management Center for Standard Materials of China Academy of Measurement Sciences.

### 2.2. Device Design and Detection Process

A high-throughput sample-in-result-out viral nucleic acid detection device was designed with Creo6.0 software, and the overall size was 440 × 411.5 × 220 mm. The shell parts of the device were 3D-printed. The device mainly comprised a detection chamber, a centrifugation drive unit, a temperature control module, an integrated optical circuit, an electronic control module, and a human–machine interface. The test tube holes in the detection chamber were arranged in an octagonal structure with a single row of 6 holes. The centrifugation drive unit included a servo motor, servo motor driver, reaction tube rack, and microcontroller unit (MCU). The temperature control module consisted of silicone heating pads, an aluminum heat sink, and temperature sensors. The integrated optical circuit included two independent fluorescence channels, FAM and ROX. The integrated optical circuit comprised a 480 nm blue LED lamp, a 570 nm green LED lamp, 4 filters (BP470/30X, BP525/20m, BP570/20X, BP612/20m), 2 monochromatic DMs (pass 570 inverse 470 and pass 612 inverse 525), and 2 4k HD 120° wide-angle cameras. LED lights were connected to a metal heat sink via a thermochemical (Arctic Silver 5, Visalia, CA, USA) and driven by a constant-current amplifier (Meanwell LDD-1000L, Guangzhou, China). The camera is controlled by MCU. The human–machine interaction, developed using the Qt GUI framework, communicated with the microcontroller through a USB serial port, with the MCU firmware written in C++ (Arduino IDE 1.8.19).

### 2.3. ASFV DNA and SARS-CoV-2 RNA Extraction

ASFV DNA templates and SARS-CoV-2 RNA were extracted and purified using a nucleic acid extraction kit according to the manufacturer’s instructions (Xi’an Tianlong Technology Co., Ltd., China). The obtained genomic DNA and RNA were detected with a Gentier32R real-time fluorescence quantitative PCR detection system (Xi’an Tianlong Technology Co., Ltd., China).

New coronavirus ribonucleic acid RNA quality control samples (high value) were purchased from the Research and Management Center for Standard Materials of China Academy of Measurement Sciences. These samples contained the important characteristic genes of the novel coronavirus: *N* gene (full-length), *E* gene (full-length), and open reading frame 1ab (*ORF1ab*) gene fragments.

### 2.4. qPCR Assay

A qPCR Nucleic Acid Test kit from Xi’an Tianlong Technology Co., Ltd. was utilized to detect ASFV according to the manufacturer’s instructions. Briefly, 15 µL of fluorescent PCR reaction solution, 5 µL of primer–probe mix, and 5 µL of DNA template were added to the reaction system to reach a total volume of 25 µL. The qPCR was digested by UDG enzyme for 2 min at 37 °C, pre-denatured by heating at 95 °C for 3 min, and then denatured at 95 °C for 15 s. The reaction was annealed at 60 °C, extended for 30 s, and circulated 45 times. The reaction was performed using a Gentier32R real-time fluorescence quantitative PCR detection system for analysis.

### 2.5. RPA and CRISPR-Cas12a Reactions

The RPA system contained a rehydration solution and reaction dry powder pellet. The 60 μL rehydration solution contained forward and reverse primer (467 nM each), 29.5 μL of RPA buffer, ribonuclease inhibitor (0.667 U/μL), MgoAc (14 mM), and 20 μL of sample release reagent–serum sample mixture. The final volume of the single-tube RPA system was 15 μL. The 10 μL CRISPR-Cas12a reaction system consisted of Cas12a (250 nM), ribonuclease inhibitor (1 U/μL), Cas12a-crRNA (500 nM), and ssDNA-FQ reporter (1.5 μM) in 1 × Cas12a buffer. The reagent components required for RPA and CRISPR were lyophilized separately, except for the template and MgoAc. The original viral sample was mixed with sample release reagents, incubated for 5 min, and then added to the inner tube. The RPA system was incubated at 39 °C for 12 min, centrifuged, mixed with CRISPR reagents, and then incubated again for 8 min.

### 2.6. RT-RPA and CRISPR-Cas12a/Cas13a Reactions

The 60 μL rehydration solution contained two pairs of forward primer (400 nM each) and two pairs of reverse primer (400 nM each) for the *ORF1ab* gene and *N* gene, 29.5 μL RPA buffer, ribonuclease inhibitor (0.667 U/μL), RNase H (0.5 U/μL), MgoAc (14 mM), and 12 μL of sample release reagent–serum sample mixture. The final volume of the single-tube RPA system was 20 μL. The 10 μL orthogonal CRISPR-Cas12a/Cas13a reaction system consisted of *ORF1ab* gene crRNA (500 nM), Cas12a (500 nM), ssDNA-FQ reporter (4.5 μM), *N* gene crRNA (500 nM), Cas13a (300 nM), ssRNA-FQ reporter (4.5 μM), ribonuclease inhibitor (2.4 U/μL), T7 RNA polymerase (8.75 U/μL), and rNTP (3.75 mM) in 1×NEB r2.1 buffer. The remaining procedure was the same as above, except for incubation times of 25 and 20 min, respectively.

### 2.7. Lyophilization of Reagents

The RPA system was lyophilized separately from the CRISPR system. The prepared RPA, without MgoAc, was further supplemented with 6% (*w*/*v*) trehalose and dispensed into precooled Eppendorf tubes. CRISPR detection reactions were prepared with 5% (*w*/*v*) PEG20000, and 6% (*w*/*v*) trehalose was added as a cryoprotectant. The loaded reagents were dropped individually into liquid nitrogen vials using automatic ball-dropping equipment and immediately placed in a freeze-dryer (−50 °C) for 1 h. This was followed by vacuum-drying at −40 °C for 10 h, −30 °C for 5 h, −10 °C for 5 h, and 20 °C for 5 h. After the reagents returned to room temperature, they were collected and stored at −20 °C.

### 2.8. ASFV Clinical Samples

ASFV clinical samples were collected from a piggery and extracted with nucleic acid reagents from Sangon Biotech (Shanghai, China). The CRISPR-Cas12a assay was performed using the proposed method. Unextracted ASFV samples were lysed with sample release reagent from Sangon Biotech at a dilution of 1:9 for 5 min at room temperature. Then, the mixture was subjected to RPA reaction and CRISPR detection. At the same time, quantitative PCR testing for the African swine fever virus was performed using an ASFV Nucleic Acid Test kit (Xi’an Tianlong Technology Co., Ltd.) according to the instruction manual.

## 3. Results and Discussion

### 3.1. Nucleic Acid Extraction-Free Technology and Lyophilization of Reagents

**Figure 2:**
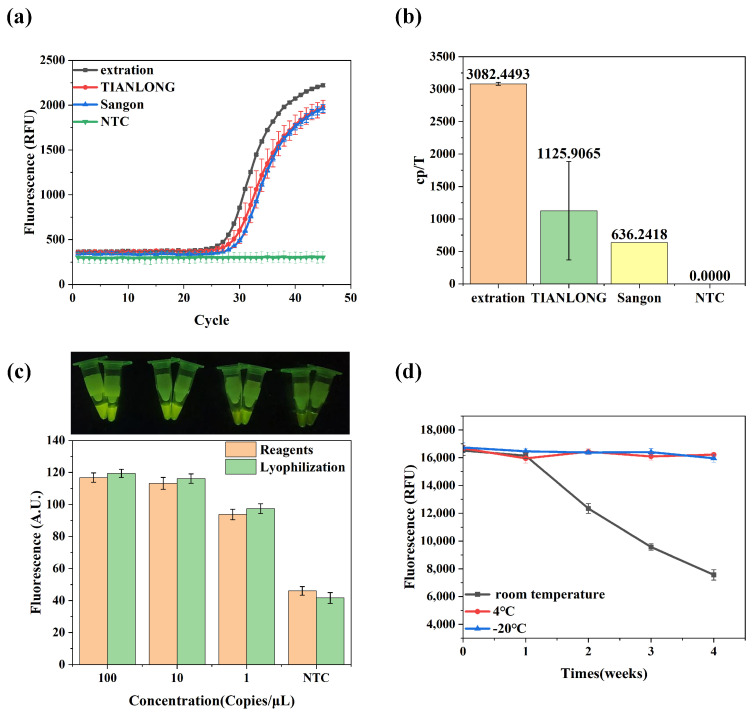
Nucleic acid extraction-free technology and lyophilization of reagents. (**a**,**b**) Sample release reagent testing: For comparison, nucleic acid extraction and two sample release reagents were used. The figures demonstrate the real-time fluorescence profile (**a**) and copy number (**b**) of the qPCR assay (NTC, nontemplate control reaction). (**c**) Lyophilized bead performance testing: 100 copies/μL, 10 copies/μL, and 1 copy/μL of ASFV plasmid were tested before and after lyophilization, and images were captured under blue LED light. To evaluate the fluorescence intensity, the fluorescence images of samples were analyzed by ImageJ2 software. (**d**) Lyophilized reagent storage testing: Difference in fluorescence of lyophilized reagents after 0, 1, 2, 3 and 4 weeks of storage at room temperature, 4 °C and −20 °C. Bar graph data presented as the mean ± SD of three experimental replicates.

To quickly disrupt the protein structure, the extraction-free technology uses an appropriate amount of protein denaturants and biochemical reagents, leading to nucleic acids release [36]. Further technical principles are detailed in Supplementary Materials S1. The technology only needs the sample to be mixed with a sample release reagent and processed at room temperature for 5 min, which can directly participate in the subsequent reaction without further purification. The released nucleic acid is directly used as a template for the amplification and detection of reactions and is paired with a constant-temperature amplification system, which enables the efficient and rapid detection of virus. To be an effective approach, sample release reagents must inactivate nucleases and infectious viral particles and must be compatible with the assay, allowing for good diagnostic performance. In order to select more effective sample release reagents, the tolerance of four different sample release reagents were tested using fluorescent RPA (Supplementary Figure S1a); the results showed that all four sample release reagents facilitated the RPA reaction. The lysis ability of each sample release reagent was further evaluated to verify the effect on the overall reaction system (Supplementary Figure S1b). The results showed that the sample release reagents of Tianlong and Sangon were more capable of lysis. For comparison with the magnetic bead method of nucleic acid extraction, the qPCR fluorescence probe method was used for their relative quantification to assess the performance of the sample release reagents (Figure 2a). The results in Figure 2b indicate that the lysis effect of the two sample release reagents was weaker than that of the magnetic bead method of nucleic acid extraction. Compared to the magnetic bead method, the average cleavage capacity of the sample release reagents from Xi’an Tianlong Technology Co., Ltd., was lower by 62%, and the average cleavage capacity of the sample release reagents from Sangon Biotech was lower by 79%. However, the sample release reagent from Sangon Biotech was chosen for the subsequent experiments due to its better stability.

In addition to extraction-free technology, reagent lyophilization technology was integrated into this study, involving separately lyophilizing the RPA and CRISPR detection components. The lyophilized beads were user-friendly, easily resolubilized, and compatible with disposable assay consumables. In our previous study, our team developed a contamination-free CRISPR-Cas13a nucleic acid assay with a detection limit of 3 copies/μL [28]. Based on this detection limit, a concentration gradient of ASFV plasmid ranging from 100 to 1 copy/μL was used for the CRISPR-Cas12a reaction to monitor the reagents’ performance before and after lyophilization. The amplification and detection reactions were conducted according to an optimized protocol, with a total reaction time of 20 min. The results revealed that when the sample concentration was within the same order of magnitude, the fluorescence intensities were comparable before and after lyophilization, and the lyophilized reagents also exhibited good sensitivity for low-copy targets (Figure 2c). In order to determine the stability of the lyophilized assay reagents at different temperatures, the lyophilized beads were stored at room temperature, 4 °C, and −20 °C for four weeks, and then the lyophilized beads were tested for activity. The experimental results are shown in Figure 2d; the lyophilization could be kept fully active for 1 week at room temperature. However, the detection effect decreased when kept at room temperature for more than a week, but the fluorescence signal could still be detected by the nucleic acid detection instrument. The lyophilized activity could be maintained for at least one month at 4 °C or −20 °C, and the detection effect did not change over time.

### 3.2. High-Throughput Sample-in-Results-Out Viral Nucleic Acid Detection Device

**Figure 3:**
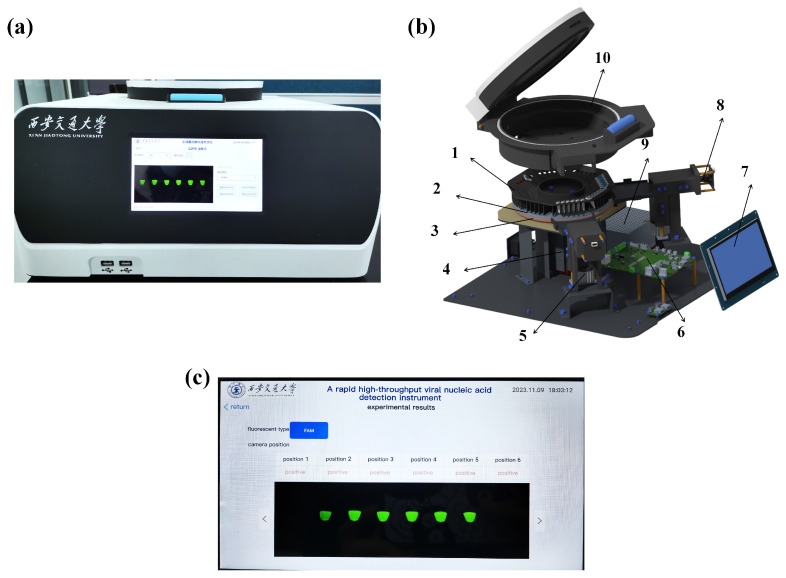
Schematic diagrams of the high-throughput sample-in-result-out viral nucleic acid detection device. (**a**) Physical diagram of the virus nucleic acid detection device. (**b**) Exploded view of the internal structure of the device: (1) sample tube rack, (2) heat sink, (3) heating pads, (4) motor, (5) LED light source, (6) circuit board, (7) HMI, (8) CCD, (9) power supply, (10) test chamber shell. (**c**) Diagram showing experimental results of the virus nucleic acid detection devices.

A high-throughput sample-in-result-out viral nucleic acid detection device was designed and customized for tube-in-tube reaction vessels. The overall physical diagram of the device is shown in Figure 3a, and each device module is shown in Figure 3b. The device components included a detection chamber, a centrifugation drive unit, a temperature control module, an integrated optical circuit, an electronic control module, and a human–machine interface (HMI). The device can adjust the temperature and integrated centrifugation, oscillatory mixing, automated fluorescence detection, result interpretation, and report output.

The centrifugation drive unit is connected to sample racks via a closed-loop stepper motor for sample mixing and centrifugation. The temperature control module is equipped with a 24 V 20 W silicone heater and a customized aluminum heat sink for efficient heating. Temperature sensors are used to monitor ambient and heater temperatures. A proportional-integral-derivative (PID) system is integrated with the MCU to maintain the ±0.5 °C temperature error. The device scans each row of sample tubes sequentially by rotating the motor, simultaneously collecting FAM and ROX fluorescence signals of 48 samples. An integrated optical circuit is employed to excite and detect the fluorescent signals, and results are displayed on an HMI (Figure 3c). The HMI allows parameter setting, real-time monitoring, and result output in a report form. All operations are implemented by the electronic control module. Supplementary Figure S2a,b validate the optical circuits with monochromatic results, and there were edge effects in the optical circuits. Supplementary Figure S2c,d reflect that the edge effect of the optical circuit did not affect the distinction between negative and positive samples.

The sample-in-results-out operation was performed using the device. First, the lyophilized CRISPR reagents were added to the outer tube, and lyophilized RPA reagents were added to an inner tube. Second, the original viral sample was mixed with the sample release reagents, incubated for 5 min, and then added to the inner tube. After closing the tube cap, the entire cannula structure was placed on a tube rack to initiate the detection phase. The automated detection phase included RPA incubation at 39 °C, centrifugation at 1200 rpm for 1 min, and three centrifugation cycles at 350 rpm with forward and reverse rotation, each lasting 5 s. Third, the sample was incubated at 39 °C for CRISPR detection. Then, the LED light and camera were activated, and the tube containing reagents on one side was photographed. The other tubes were photographed by controlling the rotating motor at a fixed angle. The results were displayed on a screen and exported through a USB port. The constructed device exhibited a high detection throughput in a short time, assaying 48 samples simultaneously within 25 min. The device was easy to use, did not require specialized personnel to operate, and reduced aerosol contamination. Compared to PCR devices, this device has greatly reduced detection time and automated sample-in-result-out testing. Supplementary Table S1 displays the cost of the device design and system development. The constructed device cost only $1923. These features make it suitable for use in communities, harbors, and other settings where simple, rapid, and high-throughput nucleic acid detection and screening are required.

### 3.3. Detection Limit and Specificity Analysis of CRISPR Detection Platforms

In order to improve the detection performance of the reaction system, each reaction condition was systematically optimized (Supplementary Materials S3). When the crRNA to the Cas12a enzyme concentration ratio was 1:1, the fluorescence signal was generated faster and with stronger intensity (Supplementary Figure S3a). Therefore, 1500 nM was chosen as the fluorescence reporter concentration, and a 39 °C reaction temperature was chosen for this study (Supplementary Figure S3b,c). Since the RPA reaction time was 12 min and the CRISPR-Cas12a detection reaction time was 8 min, the entire detection process was 20 min (Supplementary Figure S3d,e).

**Figure 4:**
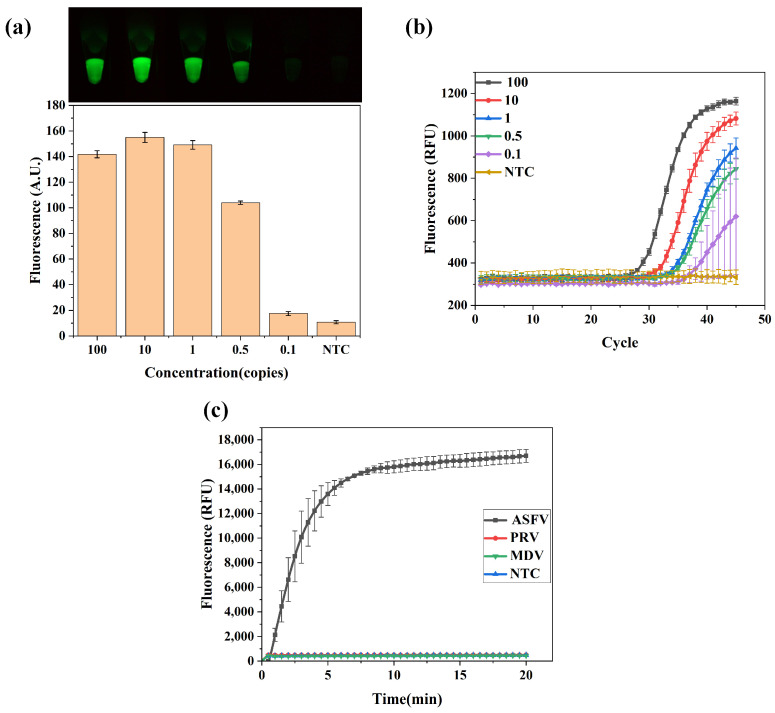
The detection limit and specificity analyses for ASFV detection. (**a**) The detection limit of the CRISPR-Cas12a assay was tested under RPA isothermal pre-amplification conditions with ASFV template of 100 copies/μL that was stepwise diluted and nontemplate control reaction (NTC). Figures were captured by the high-throughput sample-in-result-out viral nucleic acid detection device, and the fluorescence intensity was analyzed using ImageJ2 software. (**b**) The real-time fluorescence curves indicate the detection results obtained by qPCR. (**c**) RPV and MDV, double-stranded DNA viruses structurally similar to ASFV, were used for comparison with NTC and normal targets. The concentrations of 5 copies/μL of ASFV and 50 copies/μL of RPV and MDV were measured. Bar graph data presented as the mean ± SD of three experimental replicates.

Initially, the detection limit of a single CRISPR technology-based nucleic acid assay only reached 5000 copies/μL without the combination of isothermal amplification methods, which is far from meeting clinical application requirements [13]. In contrast, after adding pre-amplification methods such as RPA, the detection limit reached as low as 0.5 copies/μL [33]. The ASFV plasmid was tested at 100, 10, 1, 0.5, and 0.1 copies/μL using an optimized system. Figure 4a illustrates that the fluorescence signal was detectable when the ASFV plasmid concentration was at least 0.5 copies/μL. At 0.1 copies/μL, the fluorescence was close to that of the negative control group. Based on the amplification and the nature of the CRISPR detection itself, different concentrations of gradient samples reached the same intensity of fluorescence after a certain time. Therefore, RPA combined with the CRISPR-Cas12a assay detected ASFV plasmid at a concentration of 0.5 copies/μL. Under the same conditions, the same samples with different concentrations were analyzed by qPCR, and the detection limit was also 0.5 copies/μL (Figure 4b). These results suggest that the performance of the proposed detection method is comparable to that of qPCR. However, this method has significantly reduced detection time and could be integrated into an automatic nucleic acid detection device. Specificity is a critical metric in nucleic acid detection, ensuring the accurate recognition of target sequences [37]. The specificity of the constructed detection system was evaluated using ASFV DNA and other double-stranded DNA viruses: PRV (Pseudorabies virus) and MDV (Marek’s disease virus). Figure 4c demonstrates that the ASFV samples showed clear fluorescent signals, while other samples did not, suggesting the methods’ high specificity in detecting target sequences.

These results indicate that the CRISPR-Cas12a assay, supported by RPA pre-amplification, exhibits good detection limit and specificity and is suitable for the rapid and accurate detection of viruses such as ASFV.

### 3.4. Clinical ASFV Sample Detection

**Figure 5:**
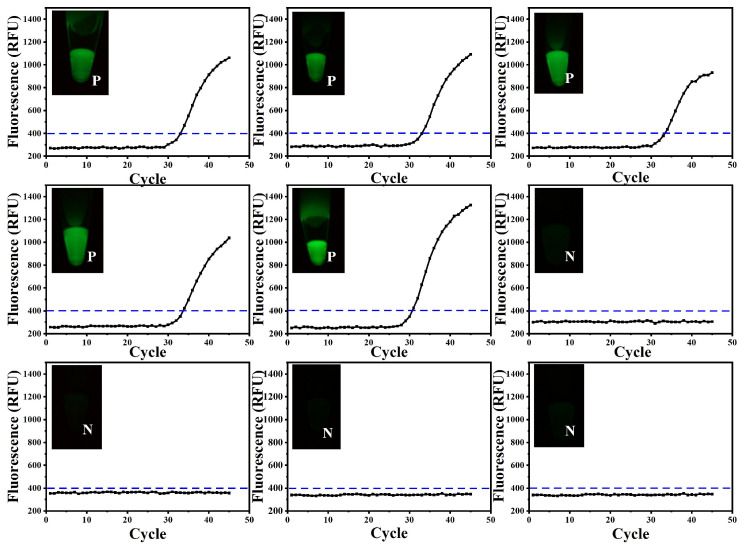
RPA-CRISPR-Cas12a assay for the detection of clinical ASFV samples. After detection by the high-throughput sample-in-result-out nucleic acid detection equipment, the fluorescence images of the endpoint were captured by the designed device. The results were compared to those of the qPCR method. Finally, the endpoint fluorescence images of the same samples were combined with the graphs.

Nine clinical ASFV samples were analyzed to verify the clinical practicability of the designed nucleic acid detection device. Among them, five were ASFV-positive and four were negative (Figure 5), showing overall agreement with the laboratory’s RT-qPCR results. The obtained results demonstrate that the CRISPR-Cas12a method, combined with a sample release reagent and lyophilized reagents, effectively detected ASFV in real animal samples. These findings reflect the method’s reliability and accuracy in practical applications in the rapid detection of diseases like African swine fever.

### 3.5. CRISPR-Cas12a/Cas13a Orthogonal Nucleic Acid Assay for SARS-CoV-2

The detection of multiple genes, biomarkers, or pathogens is used for most in vitro diagnostics [38,39,40,41,42]. Therefore, there is an increasing demand for multiplex detection systems. Ghouneimy et al. [41] established a CRISPR-Cas multiplexed diagnostic assay (CRISPRD) for cervical-cancer-causing hrHPV detection in a single reaction (one-pot assay). The assay harnesses the compatibility of AapCas12b, TccCas13a, and HheCas13a nucleases with isothermal amplification. The proposed assay successfully detected HPV16 and HPV18 and the internal control *RNase P* with a detection limit of 10 copies within 1 h. Tian et al. [42] described a highly efficient dual-gene diagnostic technique based on the orthogonal DNA/RNA collateral cleavage mechanism of the Cas12a/Cas13a system. The system could simultaneously detect the *ORF1ab* and *N* genes of SARS-CoV-2 with a detection limit of 8 copies/μL within 60 min. On this basis, the device proposed herein was improved to achieve dual-channel detection, detecting two target genes in a single tube using SARS-CoV-2 as an example. The FAM-dye ssDNA-FQ reporter and ROX-dye ssRNA-FQ reporter were used as fluorescence signals in both the CRISPR-Cas12a and CRISPR-Cas13a systems. The Cas12a-crRNA complex recognized the *ORF1ab* gene RT-RPA amplification products. The *N* gene RT-RPA amplification products were converted to RNA by T7 transcription and recognized by the Cas13a-crRNA complex. The recognition of the target-activated Cas12a enzyme and Cas13a enzyme induced two-color fluorescence emission by the orthogonal cleavage of ssDNA and ssRNA reporters. Then, the FAM and ROX emissions were simultaneously monitored by the constructed viral nucleic acid detection device.

**Figure 6:**
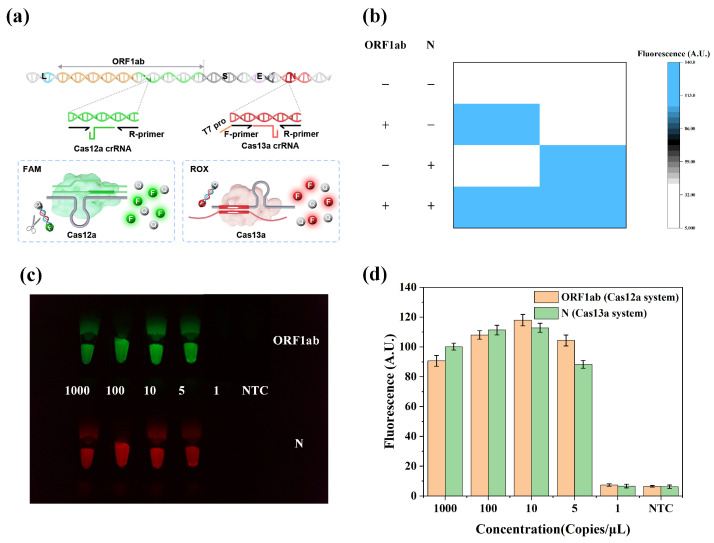
CRISPR-Cas12a/Cas13a orthogonal detection system. (**a**) Schematic diagram of the CRISPR-Cas12a/Cas13a orthogonal detection system. (**b**) Validation of orthogonal specificity. The presence of each target RNA (100 copies/μL) was confirmed by fluorescence heatmap analysis. (**c**,**d**) Detection limit assessment of CRISPR-Cas12a/Cas13a-assisted RPA for SARS-CoV-2. RNA templates were progressively diluted from 1000 copies/μL to 1 copy/μL and NTC. (**c**) Fluorescence images were captured by high-throughput sample-in-results-out viral nucleic acid detection device. (**d**) Analysis of the green and red fluorescence using ImageJ2 software. Bar graph data presented as the mean ± SD of three replicates.

Combining the nucleic acid extraction-free method, RPA, and CRISPR-Cas12a/Cas13a orthogonal nucleic acid detection, the dual-target detection of 48 samples was simultaneously achieved in less than 45 min using the viral nucleic acid detection device. We evaluated the ability of the Cas12a and Cas13a assays to analyze multiple RT-RPA products orthogonally in a single-tube system. Figure 6b illustrates that the simultaneous detection of FAM or ROX signals was only possible in the presence of two target genes. To evaluate the feasibility and stability of the method, SARS-CoV-2 quality control samples were tested using concentrations of 1000, 100, 50, 10, 5, and 1 copies/μL. The fluorescence images were captured by the designed device (Figure 6c). The results showed that the detection limit was 5 copies/μL for both the *ORF1ab* and *N* genes (Figure 6d), illustrating that SARS-CoV-2 RNA with a concentration of more than 5 copies/μL could be detected. These experimental results demonstrate that the detection limit of the CRISPR-Cas12a/Cas13a orthologous nucleic acid detection is lower than those of CRISPR-Cas12a and CRISPR-Cas13a alone. To measure the performance of the CRISPR-Cas12a/Cas13a orthogonal detection system, we analyzed 15 SARS-CoV-2-positive simulated samples and 15 SARS-CoV-2-negative simulated samples. A mixture of multiple nucleic acid standards was used to simulate those samples. Based on the analysis of 30 samples, the reproducibility of the CRISPR-Cas12a/Cas13a orthogonal detection system was 93.33% for the simultaneous detection of both genes (Supplementary Figure S4). This study confirmed the feasibility and stability of the high-throughput sample-in-results-out viral nucleic acid detection device for RPA combined with the CRISPR-Cas12a/Cas13a orthogonal detection system.

## 4. Conclusions

In this work, a high-throughput sample-in-result-out viral nucleic acid detection device was designed, aiming to achieve “sample-in-results-out” with high accuracy, high efficiency, zero contamination, and effective visualization of the entire process. A single-tube RPA-CRISPR-Cas12a assay was established, utilizing a tube-in-tube consumable, extraction-free technology, with reagent lyophilization, RPA, and CRISPR detection. The designed system simplified the operation process and avoided aerosol contamination. Combined with this assay, the constructed device could simultaneously qualitatively analyze 48 samples in 25 min, ideally suitable for nucleic acid detection in challenging conditions. The proposed method was extended to the CRISPR-Cas12a/Cas13a orthogonal detection system to realize single-tube, dual-target detection. The method’s detection limit was 0.5 copies/μL for ASFV single-gene detection within 25 min and 5 copies/μL for both the *ORF1ab* and *N* genes of SARS-CoV-2 within 45 min. These findings indicate a rapid response with a higher detection limit than the current CRISPR-based nucleic acid detection methods (Supplementary Table S2). The designed nucleic acid detection platform is highly specific, reliable, and stable for rapid qualitative detection, with 100% accuracy for single-gene detection and 93.33% accuracy for dual-target detection.

The method provides effective support for on-site detection and large-scale visual pathogen screening. It also offers a feasible solution for the clinical application of CRISPR nucleic acid detection technology. To broaden the scope of CRISPR technology application, we plan to make more advancements in our future work. Specifically, we aim to develop multiplexed identification methods that can simultaneously detect more than two targets to meet the demand for clinical diagnosis. Moreover, we will further optimize the instrument design to improve stability and durability and ensure accurate and reliable results in real-world applications.

## Figures and Tables

**Table 1:** Sequence information of the DNA oligonucleotides and crRNA.

Name	Sequence (5′-3′)
RPA-Forward primer (ASFV)	ATATGACCACTGGGGTTGGTATTCCTCCCGT
RPA-Reverse primer (ASFV)	ATCAACACCGAGATTGGCACAAGTTCGGAC
crRNA (ASFV)	UAAUUUCUACUAAGUGUAGAUCAUCAAAGUUCUGCAGCUCUUACA
ORF1ab-RPA-Forward primer (SARS-CoV-2)	AGATAATCAAGATCTCAATGGGTAACTGGGTA
ORF1ab-RPA-Reverse primer (SARS-CoV-2)	CTGCAGTTAAAGCCCTGGGTCAAGGTTAATA
N- RPA-Forward primer (SARS-CoV-2)	CCTCTAATACGACTCACTATAGGAGACGTGGTCCAGAACAAACCCAAGGAAATT
N- RPA-Reverse primer (SARS-CoV-2)	TGTGTAGGTCAACCACGTTCCCGAAGGTGTGT
ORF1ab-LbCas12a-crRNA (SARS-CoV-2)	UAAUUUCUACUAAGUGUAGAUCGGUGAUUUUCAUACAAACCA
N-LbuCas13a-crRNA (SARS-CoV-2)	GACCACCCCAAAAAUGAAGGGGACUAAAACAUGCCAAUGCGCGAC AUUCCGAAGA
ssDNA	FAM-UUAUU-BHQ
ssRNA	ROX-TTATT-BHQ

## Data Availability

The original contributions presented in this study are included in this article and Supplementary Material; further inquiries can be directed to the corresponding author.

## Supplementary Materials

The following supporting information can be downloaded at: https://www.mdpi.com/article/10.3390/bios14110549/s1, Supplementary S1. Performance comparison of sample release reagents: Figure S1: Performance comparison of sample release reagents; Supplementary S2. Optical testing of device: Figure S2: Device optical path edge effect detection; Supplementary S3. Optimization of CRISPR analysis: Figure S3: CRISPR reaction analysis optimization. Supplementary S4. Materials cost analysis of the device: Table S1. Materials cost analysis of the device. Supplementary S5. Comparison of CRISPR-based methods: Table S2. Comparison of CRISPR-based methods. Supplementary S6. Performance testing of the orthogonal system: Figure S4. Performance testing of the CRISPR-Cas12a/Cas13a orthogonal detection system for the detection of SARS-CoV-2. References [13,14,15,16,17,42,43] are cited in the Supplementary Materials.
