# Supplementary material for: A Novel High-Throughput Sample-in-Result-Out Device for the Rapid Detection of Viral Nucleic Acids
Source: Biosensors (Basel). 2024 Nov 13;14(11):549. doi: 10.3390/bios14110549 (PMC11591587; doi:10.3390/bios14110549)
Supplement: Supplementary file 1 [file biosensors-14-00549-s001.zip › biosensors-3194796-supplementary.pdf]

Supplementary Materials for

A novel high-throughput sample-in-result-out device for rapid detection of viral nucleic acids

Fangning Wang[1], Fei Hu[1]*, Yunyun Zhang[1], Xichen Li[1], Qin Ma[1], Xincheng Wang[1], Niancai Peng[1]

[1] State Key Laboratory for Manufacturing Systems Engineering, School of Instrument Science and Techonology, Xi'an Jiaotong University, Xi'an 710054, China.

Contents

S1. Performance comparison of sample release reagents
S2. Optical testing of device
S3. Optimization of CRISPR analysis
S4. Materials cost analysis of the device
S5. Comparison of CRISPR-based methods
S6. Performance testing of the orthogonal system

S1. Performance comparison of sample release reagents

The sample release reagents were composed of a cationic surfactant, alginate, polyethylene glycol, Tris-HCl, EDTA, Triton X-100, and other components. The cationic surfactant (dodecyldimethylbenzylammonium chloride or bromide) exhibited low toxicity and strong adsorption capacity to bacterial surfaces and denatured protein, released nucleic acid, and effectively cleaved viral envelopes and capsids. Alginate and polyethylene glycol (PEG) created a protective layer around nucleic acids (RNA or DNA), stabilizing their integrity. Tris-HCl maintains pH, ensuring compatibility with subsequent amplification steps. Triton X-100 and dodecyldimethylbenzylammonium chloride emulsified fats and alters cell permeability. EDTA chelates metal ions, reducing DNA or RNA enzyme activity. The nucleic acid release system involved mixing sample release reagents with serum samples in a specific ratio, using protein denaturant and biochemical reagents to disrupt protein structure and release nucleic acids rapidly. The released nucleic acids can be directly used in subsequent RPA reactions after mixing.

The tolerance of sample release reagents was tested using the ASFV chain-interactive nucleic acid amplification reaction system (fluorescent) from Xi'an Tianlong Technology Co., Ltd, Sangon Biotech (Shanghai, China), and Suzhou Senta Gene Technology Co., Ltd. PC samples comprised 100 copies/μL of the ASFV virus plasmid. The 5 μL template contained 1 μL ASFV virus sample and the corresponding proportion of sample release reagents and was replenished to 5 μL using ddH$_2$O. Experimental results demonstrated that the sample release reagent from each company showed a positive impact on the reaction system (Figure S1a). The cleavage capacity of sample release reagents was further assessed. ASFV serum samples were mixed with sample release reagents as per the instructions of each company using ASFV virus plasmid at a concentration of 100 copies/μL as control and left to stand for 5 min for the RPA and CRISPR-Cas12a reactions. Results indicated that the sample release reagent from Xi'an Tianlong Technology Co. and Sangon Biotech (Shanghai, China) companies exhibited superior performance (Figure S1b).

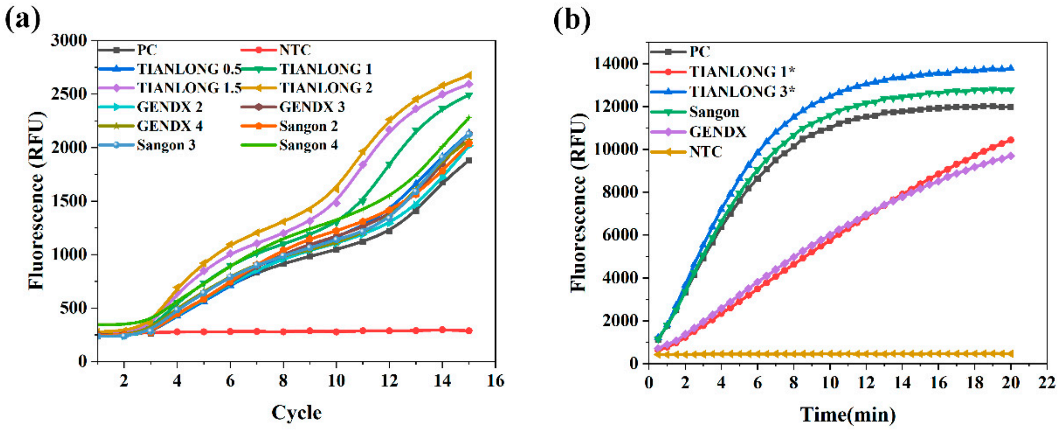

Figure S1. Performance comparison of sample release reagents. a) The effect of different sample release reagents concentrations from each company using fluorescent RPA. b) The lysing ability of sample release reagents from each company tested using the same serum sample. PC samples

comprised 100 copies/μL of the ASFV virus plasmid.

S2. Optical testing of device

After assembling the high-throughput sample-in-result-out viral nucleic acid detection device, the feasibility of the optical circuit was verified using monochromatic results. The same positive sample was sequentially placed in six tube rack holes on one side of the octagonal tube tray, photographed, and recorded individually (Figure S2a). Each test tube's brightness was compared, and the fluorescence intensity was quantified using ImageJ software. The light intensity at both ends of the ROX pathway was lower compared to the middle part due to the edge effect, with the weakest fluorescence observed at wells 1 and 6, and the strongest fluorescence at wells 3 and 4. Conversely, in the FAM channel, the intensity of the leftmost test tube was lower than the middle part, with the weakest fluorescence at well 1 and the strongest at well 4 (Figure S2b).

To assess the edge effect of the optical circuit on the differentiation of negative and positive results, the same positive samples were placed into wells 1 and 6 with the weakest light intensity, photographed, and recorded individually. Simultaneously, the same negative sample was placed into wells 3 and 4 with the strongest light intensity (Figure S2c). To quantify the fluorescence value, the images were processed using ImageJ software. Positive samples exhibited noticeable fluorescence signals in the weakest 1 and 6 wells, while negative samples did not show fluorescence signals in the strongest 3 and 4 wells (Figure S2d). The results reflected that the edge effect of the light path did not influence the differentiation between negative and positive samples.

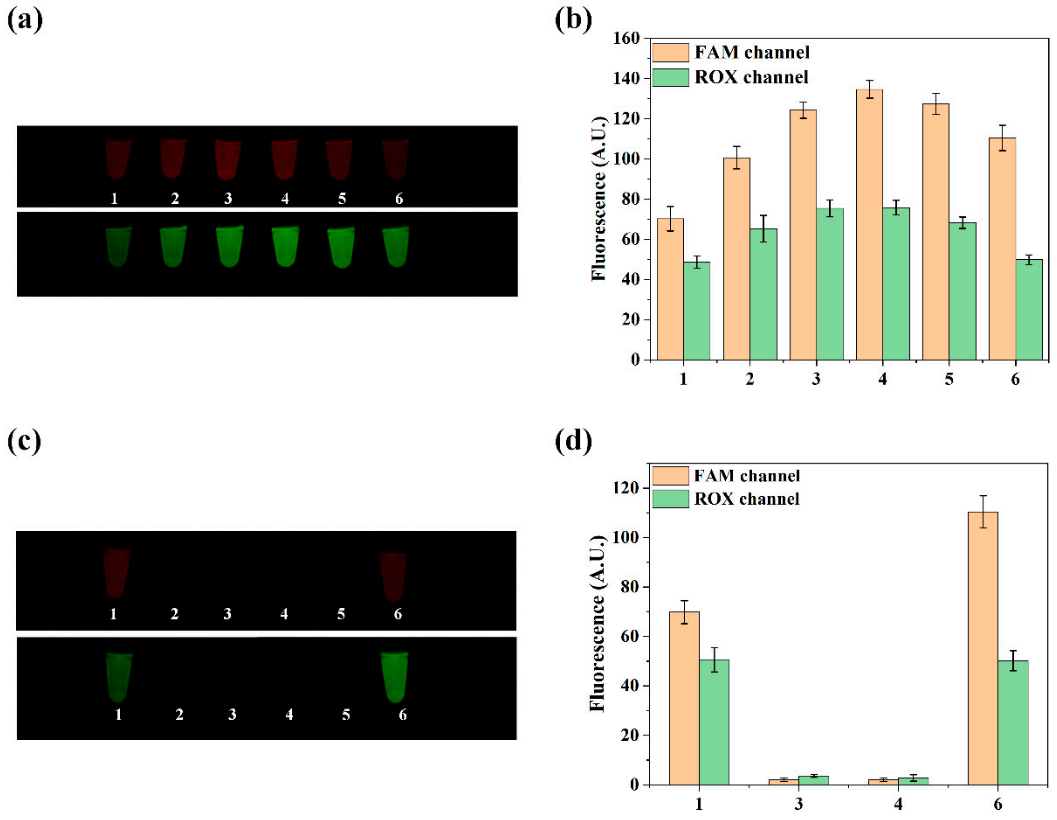

Figure S2. Device optical path edge effect detection. a) Fluorescence image taken by placing the same positive sample into the six wells on one side of the octagonal tube tray one at a time. b) Analysis of the green and red fluorescence images using the ImageJ software. c) Fluorescence images taken by simultaneously placing the same positive sample into wells 1 and 6 and the same negative sample into wells 3 and 4. d) Analysis of the green and red fluorescence images using the ImageJ software. Bar graph data represented as the mean ± SD of three experimental replicates.

S3. Optimization of CRISPR analysis

Since the reaction efficiency of RPA and CRISPR-Cas12a assay directly affect the sensitivity, this research systematically investigated each reaction condition to optimize the analytical performance.

A single-variable approach was used to optimize the test conditions. We set up different concentration ratio gradients (1:10, 1:5, 1:2, 1:1, 2:1, 5:1, 10:1) to explore the optimal concentration ratio while fixing the Cas12a enzyme concentration at 100 nM. To explore the optimal ssDNA concentration, different concentration gradients (100 nM, 500 nM, 1000 nM, 1500 nM, 2000 nM, 2500 nM, and 3000 nM) were set up, and nucleic acid detection experiments were performed. To investigate the specific optimal temperature when it exceeds this range, different temperature gradients (32°C, 35 °C, 37 °C, 38 °C, 39 °C, 40 °C, 41 °C, 45 °C, and 49 °C) were set up, and crRNA to Cas12a enzyme ratio was controlled at 1:1 using ssDNA concentration of 1500 nM. The RPA reaction time and CRISPR detection time were 20 min.

The experimental results showed that when the crRNA to the Cas12a enzyme

concentration ratio was 1:1, the fluorescence signal was generated faster with stronger intensity (Figure S3a). When the fluorescent reporter concentration was higher than 1500 nM, further increase in concentration had little effect on the fluorescence signal generation rate (Figure S3b). Therefore, 1500 nM was chosen as the fluorescent reporter concentration for this study. An obvious fluorescence signal was generated in the temperature range of 37 °C to 41 °C, and the signal was weak below or above this range (Figure S3c). Therefore, the reaction temperature of 39 °C was chosen for subsequent studies.

Then, the shortest reaction times for RPA and CRISPR detection were explored separately to optimize the overall detection process. The fluorescence curves indicated that the RPA reaction was saturated at approximately 12 min, as shown in Figure.S3d. Therefore, the RPA amplification reaction time was selected as 12 min. The CRISPR reaction was carried out at different times after initiating the RPA reaction for 10, 12, 15, and 18 min to determine the optimal time for the CRISPR reaction and the best combination of the two reaction times. The fluorescence analysis curves revealed that the RPA reaction time positively correlated with the fluorescence signal enhancement rate and the final fluorescence intensity; however, the enhancement rate decreased significantly after 12 min (Figure S3e). Additionally, the fluorescence signal intensity gradually increased as the CRISPR reaction progressed at a constant RPA reaction time; however, the enhancement rate decreased significantly after 8 min (Figure.S3e). Therefore, the RPA amplification reaction time was set to 12 min, and the CRISPR-Cas12a detection reaction time was 8 min, allowing for the completion of the detection process in 20 min.

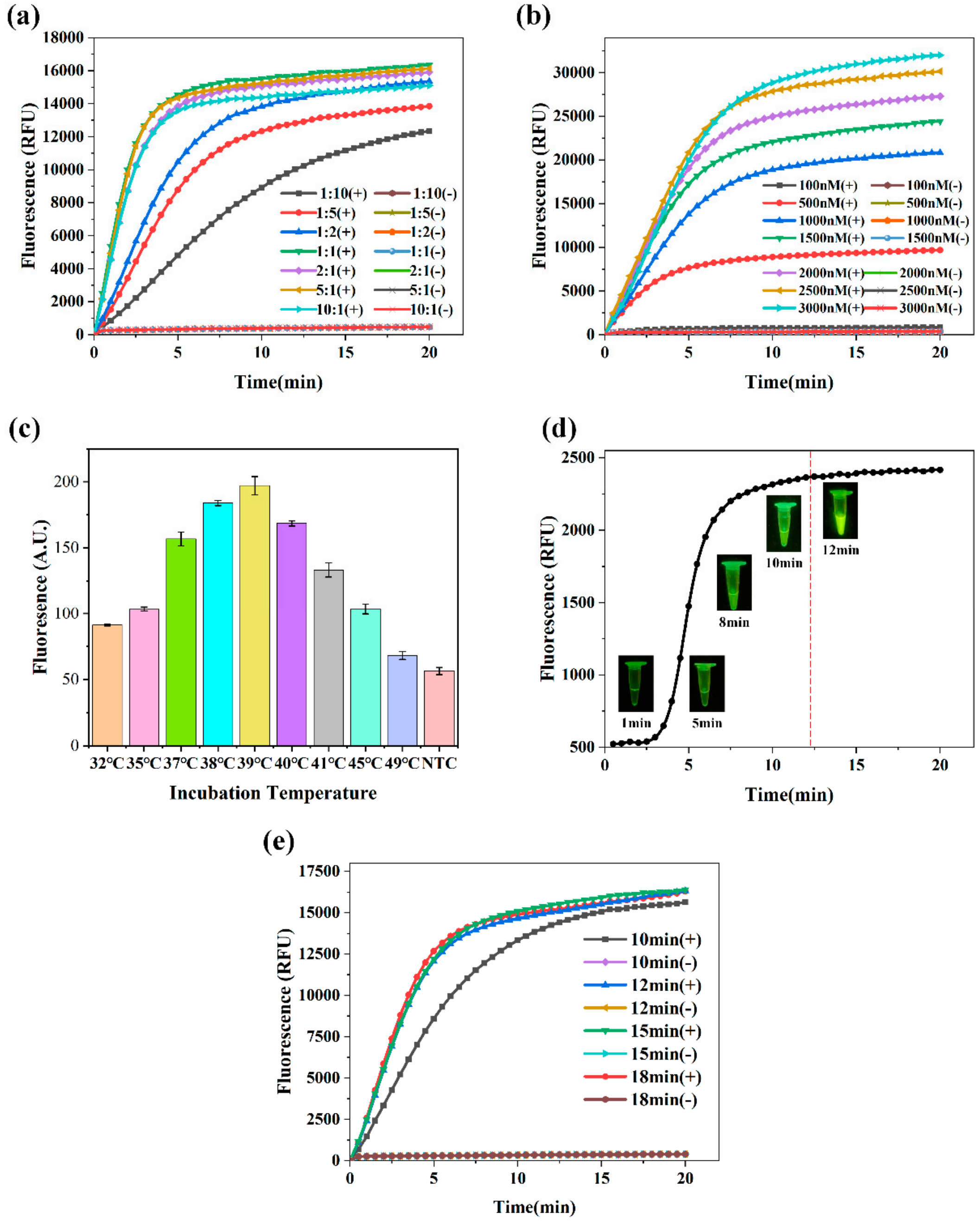

Figure S3. CRISPR reaction analysis optimization. a) The crRNA to Cas12a enzyme concentration ratio and controlling of Cas12a enzyme concentration at 100nM. b) ssDNA concentration optimization, controlling the crRNA and Cas12a enzyme concentration at 100nM. c) Temperature optimization, controlling the crRNA and Cas12a enzyme concentration 100 nM and ssDNA concentration at 1500 nM. quantified fluorescence values were analyzed using ImageJ software. Bar graph data represented as the mean ± SD of three experimental replicates. d) Real-time fluorescence curves of RPA after adding fluorescent dyes. e) Real-time fluorescence curves of different RPA and CRISPR assay times, controlling the crRNA and Cas12a enzyme concentration at 100nM and ssDNA at 1500 nM.

S4. Materials cost analysis of the device

Table S1. Materials cost analysis of the device.

device components	Parts name	Type specification	Qty	Cost ($ USD)
Mechanical structural components	Shell<br>Sample tube rack<br>All fixing block	3D-Printed.	1	1081
optical circuit components	CCD	CMOS, IMX317 chip (1/2.5inch, length and width 6.1x4.524mm, resolution 3840x2160, pixel unit 1.62x1.62μm), lens 12mm.	2	120
	LED light source	blue 480-485nm, 5W.<br>green 565-575nm, 5W.	2	14
	Dichroic Mirror	LP505dc, 50x25mm.<br>LP590dc, 50x25mm.	2	114
	Excitation filter	BP470/30X, 20x1.0 mm.<br>BP570/20X, 20x1.0 mm.	2	94
	Emission filter	BP525/20m, 15x1.0 mm.<br>BP612/20m, 15x1.0 mm.	2	74
Electrical components	HMI	MAKEBIT-7-600TN	1	43
	Motor	57CME12X-BZ	1	11
	Power supply	NET-200-D	1	9
	Circuit board		1	342
Temperature control components	Heating pads	RBN-GJ-002	1	7
	Temperature sensors	PT100	2	14
Total cost	1923			

S5. Comparison of CRISPR-based methods

Table S2. Comparison of CRISPR-based methods.

	Time	Throughput	Sensitivity	Specificity	Accuracy	References
qPCR	1.5-4 h	/	1-10 copies/reaction	High	100%	[1]
SHERLOCK	0.5-1 h	1	2 aM	High	100%	[2]
HOLMES	1 h	1	10 aM	High	100%	[3]
DETECTR	1-2 h	1	1 aM	High	100%	[4]
CRISPR/Cas12a-NER	45 min	1	10 copies/μL	High	100%	[5]
CORDSv2	40 min	5	6 copies/μL	High	100%	[6]
Orthogonal CRISPR	1 h	1	8 copies/μL	High	100%	[7]
RPA-CRISPR/Cas12a	25 min	48	0.5 copies/μL	High	100%	this article

S6. Performance testing of the orthogonal system

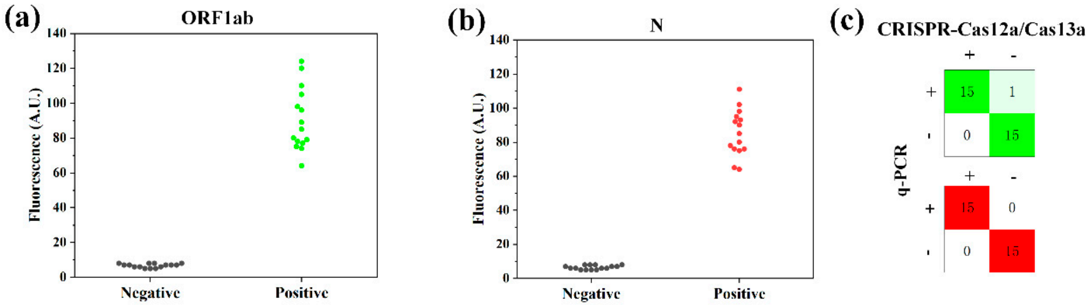

Figure S4. Performance testing of the CRISPR-Cas12a/Cas13a orthogonal detection system for the detection of SARS-CoV-2. a) Scatterplot analysis showing the fluorescence values of the *ORF1ab* gene for 15 negative and 15 positive samples detected by the CRISPR-Cas12a/Cas13a orthogonal detection system in the FAM channel. b) Scatterplot analysis showing the fluorescence values of the *N* gene for 15 negative and 15 positive samples detected by the CRISPR-Cas12a/Cas13a orthogonal detection system in the ROX channel. c) Consistency between the CRISPR-Cas12a/Cas13a orthogonal detection and RT-qPCR results.

References

1. Kubina, R.; Dziedzic, A., Molecular and Serological Tests for COVID-19. A Comparative Review of SARS-CoV-2 Coronavirus Laboratory and Point-of-Care Diagnostics. *Diagnostics* **2020,** *10* (6). https://doi.org/10.3390/diagnostics10060434

2. Gootenberg, J. S.; Abudayyeh, O. O.; Lee, J. W., et al., Nucleic acid detection with CRISPR-Cas13a/C2c2. *Science* **2017,** *356* (6336), 438-+. https://doi.org/10.1126/science.aam9321

3. Li, S. Y.; Cheng, Q. X.; Wang, J. M., et al., CRISPR-Cas12a-assisted nucleic acid detection. *Cell Discovery* **2018,** *4*. https://doi.org/10.1038/s41421-018-0028-z

4. Chen, J. S.; Ma, E. B.; Harrington, L. B., et al., CRISPR-Cas12a target binding unleashes indiscriminate single-stranded DNase activity. *Science* **2018,** *360* (6387), 436-+. https://doi.org/10.1126/science.aar6245

5. Wang, X.; Zhong, M.; Liu, Y., et al., Rapid and sensitive detection of COVID-19 using CRISPR/Cas12a-based detection with naked eye readout, CRISPR/Cas12a-NER. *Sci Bull (Beijing)* **2020,** *65* (17), 1436-1439. https://doi.org/10.1016/j.scib.2020.04.041

6. Lin, C.; Chen, F.; Huang, D., et al., A universal all-in-one RPA-Cas12a strategy with de novo autodesigner and its application in on-site ultrasensitive detection of DNA and RNA viruses. *Biosens Bioelectron* **2023,** *239*, 115609. https://doi.org/10.1016/j.bios.2023.115609

7. Tian, T.; Qiu, Z. Q.; Jiang, Y. Z., et al., Exploiting the orthogonal CRISPR-Cas12a/Cas13a trans-cleavage for dual-gene virus detection using a handheld device. *Biosensors & Bioelectronics* **2022,** *196*. https://doi.org/10.1016/j.bios.2021.113701
